# The 21st Century Cures Act and Multiuser Electronic Health Record Access: Potential Pitfalls of Information Release

**DOI:** 10.2196/34085

**Published:** 2022-02-17

**Authors:** Simone Arvisais-Anhalt, May Lau, Christoph U Lehmann, A Jay Holmgren, Richard J Medford, Charina M Ramirez, Clifford N Chen

**Affiliations:** 1 Department of Hospital Medicine University of California San Francisco San Francisco, CA United States; 2 Department of Laboratory Medicine University of California San Francisco San Francisco, CA United States; 3 Division of Developmental and Behavioral Pediatrics Department of Pediatrics University of Texas Southwestern Medical Center and Children's Medical Center Dallas Dallas, TX United States; 4 Department of Pediatrics University of Texas Southwestern Medical Center Dallas, TX United States; 5 Department of Data Sciences and Bioinformatics University of Texas Southwestern Medical Center Dallas, TX United States; 6 Department of Medicine, Center for Clinical Informatics and Improvement Research University of California San Francisco San Francisco, CA United States; 7 Division of Infectious Disease Department of Internal Medicine University of Texas Southwestern Medical Center Dallas, TX United States; 8 Division of Pediatric Gastroenterology, Hepatology, and Nutrition Department of Pediatrics University of Texas Southwestern Medical Center and Children's Medical Center Dallas Dallas, TX United States; 9 Division of Hospital Medicine Department of Pediatrics University of Texas Southwestern Medical Center and Children's Medical Center Dallas Dallas, TX United States

**Keywords:** 21st Century Cures Act, Open Notes, Information Blocking, multiuser EHR access, proxy EHR access, adolescent Health, health IT Policy, information technology, cures act, electronic health record, electronic health information, health information, patient care

## Abstract

Although the Office of The National Coordinator for Health Information Technology’s (ONC) Information Blocking Provision in the Cures Act Final Rule is an important step forward in providing patients free and unfettered access to their electronic health information (EHI), in the contexts of multiuser electronic health record (EHR) access and proxy access, concerns on the potential for harm in adolescent care contexts exist. We describe how the provision could erode patients’ (both adolescent and older patients alike) trust and willingness to seek care. The rule’s preventing harm exception does not apply to situations where the patient is a minor and the health care provider wishes to restrict a parent’s or guardian’s access to the minor’s EHI to avoid violating the minor’s confidentiality and potentially harming patient-clinician trust. This may violate previously developed government principles in the design and implementation of EHRs for pediatric care. Creating legally acceptable workarounds by means such as duplicate “shadow charting” will be burdensome (and prohibitive) for health care providers. Under the privacy exception, patients have the opportunity to request information to not be shared; however, depending on institutional practices, providers and patients may have limited awareness of this exception. Notably, the privacy exception states that providers cannot “improperly encourage or induce a patient’s request to block information.” Fearing being found in violation of the information blocking provisions, providers may feel that they are unable to guide patients navigating the release of their EHI in the multiuser or proxy access setting. ONC should provide more detailed guidance on their website and targeted outreach to providers and their specialty organizations that care for adolescents and other individuals affected by the Cures Act, and researchers should carefully monitor charting habits in these multiuser or proxy access situations.

## Introduction

“*Primum non nocere*” (“*First, do no harm*”) or nonmaleficence is a fundamental principle taught to every health care provider. It suggests that before applying any medical intervention, one needs to consider the potential negative effects on the patient. In this piece, we examine the potential for patient harm by the Office of The National Coordinator for Health Information Technology’s (ONC) Information Blocking Provision in the Cures Act Final Rule and the additional burden that health care providers, those who provide patient care and provide documentation in the electronic health record, will now face when documenting sensitive information.

On December 13, 2016, the 21st Century Cures Act (hereinafter referred to as the “Cures Act”) was signed into law with the intent to “accelerate the discovery, development, and delivery of 21st century cures, and for other purposes” [1]. The Act defined electronic health record (EHR) interoperability, addressed health information technology certification requirements, and prohibited information blocking—the practice that prevents or interferes with those with permission to access electronic health information (EHI) [2]. As the federal entity coordinating efforts to implement health information technology and exchange EHI, ONC, a division within the US Department of Health and Human Services (HHS) [3], developed the Cures Act Final Rule to direct the implementation of the Cures Act legislation [4].

## The ONC Cures Act Final Rule

The stated goal of the ONC Cures Act Final Rule is to empower patients to interact “with their health record in a modern health IT economy” [4]. ONC postulated that “putting patients in charge of their health record is a key piece of patient control in healthcare and patient control is at the center of HHS’s work towards a value-based healthcare system.” The Cures Act Final Rule also encourages innovations in health care technology and hopes to deliver the following:

- Transparency on cost and outcomes of care
- Competitive options in obtaining medical care
- Convenient access to medical records using smartphone apps
- Innovation and choice for patients, physicians, hospitals, payers, and employers through an app-based economy [4]

The Cures Act Final Rule promotes interoperability across EHR vendors through the adoption of data exchange standards and calls upon the health care information technology (IT) industry to adopt standardized application programming interfaces through specified Conditions of Certification. Additionally, ONC aims to increase patients’ access to their EHI through minimizing measures that block patient access to information [5-7].

## The Information Blocking Provision

The Information Blocking Provision of the Cures Act Final Rule mandates that patients have unfettered, free access to their EHI, and provides clear requirements for compliance by health care providers, institutions, health information exchanges, and EHR vendors [8].

**Figure 1:**
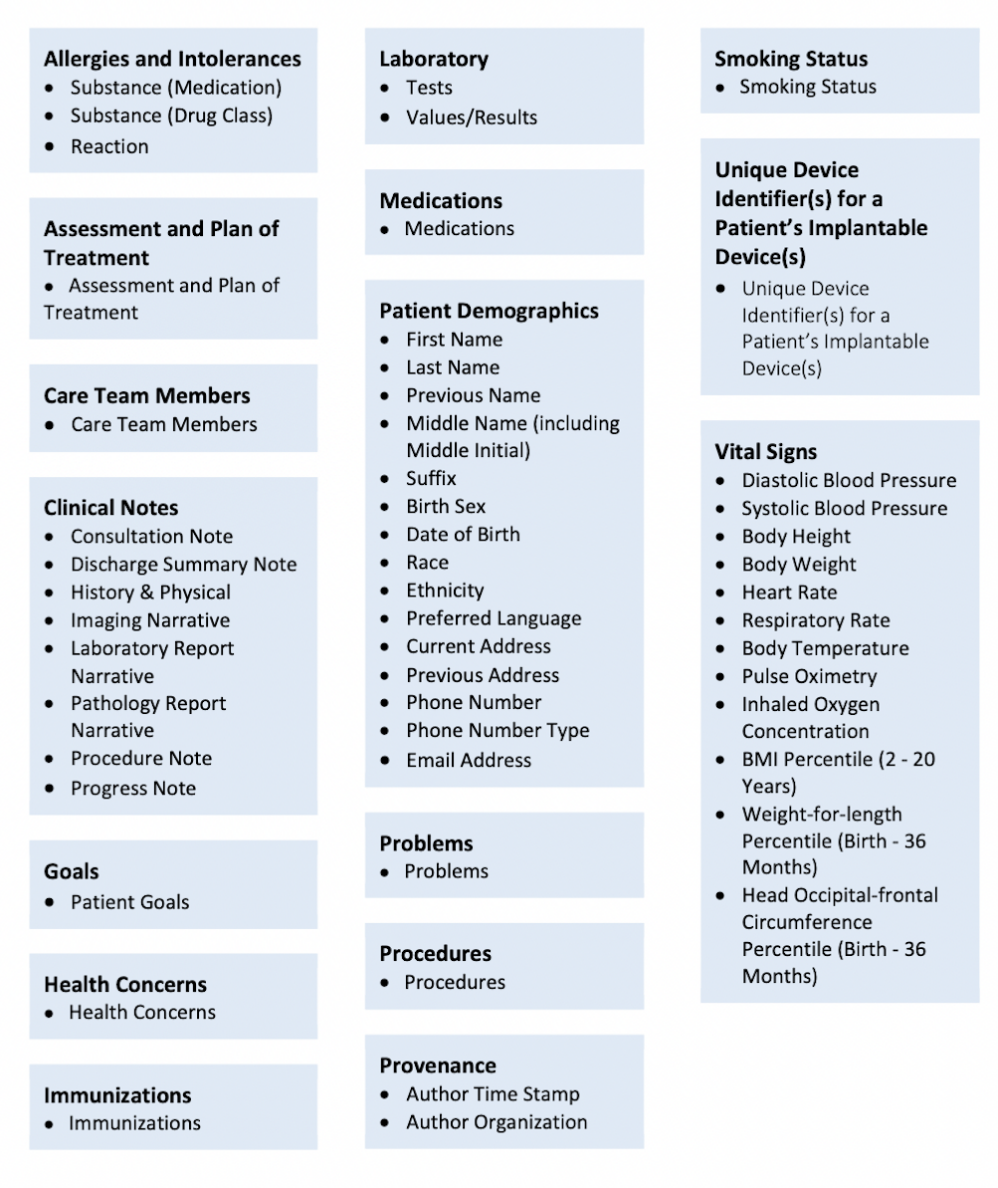
Elements of the United States Core Data for Interoperability [11].

The spirit of the Information Blocking Provision is similar to that of the OpenNotes movement, which over the past decade has been adopted by several health care institutions across the United States, Canada, and Sweden and provides patients with near immediate and full access to their EHI [9,10]. The Information Blocking Provision requires that patients have access to parts of their EHI defined by the United States Core Data for Interoperability (Figure 1) by April 5, 2021, with eventual expansion to all EHI by October 6, 2022 [11-13]. Of note, patients have had the right to access their medical record since the implementation of the Health Insurance Portability and Accountability Act (HIPAA). The Cures Act final rule does not increase the type of health information that patients and families can access, it only facilitates automatic release via patient portals and easier access electronically.

The Information Blocking Provision includes a means to report violations and enforcement options. ONC encourages anyone who experiences or observes information blocking by any health care provider, health IT developer, certified health IT, health information network, or information exchange to share their concerns through an information blocking portal on ONC’s website [11]. Health IT developers, health information networks, and health information exchanges can be subject to civil monetary penalties of up to US $1,000,000 per violation [14]. Health care providers found to have committed information blocking will also be subjected to penalties that are to be determined [14].

## Exceptions to the Information Blocking Provision

The Information Blocking Provision defines eight exceptions that do not constitute information blocking [15]. The preventing harm exception and the privacy exception are applicable to the documenting health care provider.

The preventing harm exception stipulates that provided certain conditions are met, a health care provider can prevent the access to a patient’s EHI if it is “reasonable and necessary to prevent harm to a patient or another person” [15]. Key conditions include that the health care provider must reasonably believe that preventing access to a patient’s EHI will significantly reduce a risk of substantial harm, and that the interference is no broader than necessary. The patient has the right to request a review of an individualized determination of risk of harm [16].

According to ONC’s guidance in the “Information Blocking Frequently Asked Questions,” the “Preventing Harm” exception does not apply to situations where the patient is a minor and the health care provider wishes to restrict a parent or legal representative’s access to the minor’s EHI to avoid violating the minor’s confidentiality and destroying the trust between the youth and the health care provider [12]. This lack of applicability in the case of adolescent confidentiality stands in tension with principles outlined by experts in the design and implementation of EHRs [17], endorsed by the American Academy of Pediatrics and Society of Adolescent Health And Medicine [18,19]. The concern over the implication of the Cures Act on adolescent confidentiality has been noted in the literature [20]. The premise underlying confidential care encourages adolescents to communicate with health care providers about sensitive topics such as sexual and reproductive health and substance abuse without the fear that their parents or guardians will have access to this information. Confidentiality on certain health care problems facilitates obtaining medical care that adolescents might forgo if information were shared with others. In the context of providing confidential care, the Cures Act’s broad focus on patient EHI access may cause a trade-off with patient–provider relationships, trust, and nonphysical types of harm. The text of the Final Rule specifically states that the desire to maintain confidentiality and to protect patient–provider relationships is insufficient to prevent the release of sensitive information. In certain multiuser access cases, this may erode the patient’s control over his/her information instead of increasing control. It is worth noting that HIPAA and the Cures act defer to the state laws that grant adolescents the ability to consent for certain conditions. While it is challenging to keep track of each state’s individual and varied confidentiality laws that result in 56 (one for each state and territory) different legal requirements for users of pediatric EHRs, these laws do provide clear legal backing to protect adolescent confidentiality.

The other exception to the Cures Act Final Rule’s patient access provision immediately relevant to health care providers is the privacy exception. Under this exception, interfering with access to EHI is deemed not to be information blocking when the intent is to protect the patient’s privacy. These exceptions, listed in Textbox 1, are included by ONC to comply with HIPAA and other state privacy regulations and allow patients the opportunity to request information not be shared. Depending upon institutional practices, providers and patients may have limited awareness of this exception. Notably, the privacy exception states that providers cannot “improperly encourage or induce a patient’s request to block information” [21]. This stipulation affects a provider’s ability to guide patients for fear of being found in violation of the Information Blocking Provision and fined. Institutional policies and procedures will affect the implementation and management of this exception. Providers may not be aware of the procedure for patients to request their information not be shared during an encounter. Depending upon when the patient places the request (eg, before, during, or after an encounter), the institution may not be able to fulfill the request in a timely manner relative to the immediacy of information being released. Additionally, the privacy exception does not clearly describe if and how a patient can block individual pieces of data (data segmentation) instead of all data. The exception only describes how patients can request to block access and can request to regain access. The interpretation and implementation of this exception is left to the institution and provider and, given the complex nature of the exception, necessitates deference to informatics expertise and legal resources with experience in state and federal privacy laws and statutes for interpretation and use.

**Privacy exceptions to information blocking.**

**Exceptions:**

- More stringent state or federal preconditions to exchange is not met 
- Information technology developer is not covered by the Health Insurance Portability and Accountability Act Privacy Rule 
- Inability to validate a requester’s right to access 
- The individual requests the information not to be shared 

## Limitations to the Information Blocking Provision: Multiuser or Proxy Access

### Overview

The OpenNotes initiative has been shown to potentially increase patient activation, engagement, satisfaction, trust, and safety, and to improve the patient–physician relationship [22-25]. However, concern exists that the Information Blocking Provision will result in damaging breaches of confidentiality for cohorts of patients when parents or legal representatives are provided multiuser or proxy access to EHI [26,27]. In circumstances where EHI is made available within a web-based portal with multiuser or proxy access, the information could compromise the confidentiality of the patient, parent, or legal representatives and damage the relationship between the health care provider, patient, parent, or legal representative. The breach of confidentiality may occur bidirectionally as a caregiver may share information with a provider, which could be shared back with the patient. One recent study highlighted another area of concern: when guardians access an adolescent patient’s portal account. The study revealed that the estimated prevalence of guardian access could be as high as 76% of adolescent accounts and also showed a relatively low rate of proxy account creation [28]. When adolescents had their own portal account, proxy accounts for adolescent patients were created in only 0.3%-10% of cases [28]. The reality that many portal accounts are used and managed by guardians must be taken into consideration for adolescent patients who, in the context of their care setting, may lack the autonomy to prevent their guardians from accessing their personal patient portals.

### Pediatric and Adolescent Patients

Prior to the Information Blocking Provision, pediatric institutions participating in the OpenNotes movement had addressed the concern for violating confidentiality and damaging relationships by blocking all clinical notes from several clinics including adolescent, gynecology, psychiatry, substance abuse, and the child protection team [29]. Although the Cures Act Final Rule explicitly states that maintaining confidentiality and protecting relationships is not sufficient to prevent the release of sensitive information, the effects of releasing this information on patients, their parents or legal representatives, and the patient–provider relationship cannot be underestimated and are concerning to adolescent medicine providers and other health care providers who care for youth [30].

### The Adolescent–Health Care Provider Relationship

There are many situations that do not fit the “Preventing Harm” exception where adolescent patients may be adversely affected when their private information is accessed by others (Table 1). For example, an adolescent female with concerns for a sexually transmitted infection (STI) such as *Neisseria gonorrhoeae* or *Chlamydia trachomatis* may avoid seeking medical care to avoid repercussions or stigma if she knew her parents would have access to this information. This untreated STI could progress to pelvic inflammatory disease, a more serious infection, which may require hospitalization and intravenous antibiotic administration and could affect future fertility. Prior research has shown that 59% of surveyed females younger than 18 years would “stop using all sexual healthcare services, delay testing or treatment for HIV or other STDs, or discontinue use of specific (but not all) sexual healthcare services if their parents were informed they were seeking prescribed contraceptives” [31]. The concern for loss of confidentiality extends to other sensitive topics including mental health, substance use, gender identity, and sexual orientation and may conflict with federal and state laws.

**Table 1:** Hypothetical scenarios for potential harm related to either lack of clarity of the laws, technical limitations regarding the release of electronic health information, or a combination of both.

At risk for harm	Third party receiving information	Domain	Mode of disclosure	Consequence
Patient	Parents or guardian	Mental health	Patient portal	Avoiding care or deterioration
Patient	Parents or guardian	Substance use	Patient portal	Avoiding care, overdose, or continued addiction
Patient	Parents or guardian	Sexual history or reproductive health	Patient portal	Avoiding care, complications from sexually transmitted infection, or infertility
Patient	Parents or guardian	Gender management or identity	Patient portal	Avoiding care, delay in gender reassignment, or psychological impact
Patient	Parents, guardian, or abuser	Violence or abuse (physical or sexual)	Patient portal	Avoiding care, continued abuse, complications, or death
Patient	Parents or guardian	Complex social situations	Patient portal	Avoiding care or delayed care
Patient	Parents or guardian	Neglect	Patient portal	Avoiding care, delayed care. or continued neglect
Child/Adolescent	Parents or guardian	Foster or custody issues	Patient portal	Avoiding care, delayed care, or family strife
Child/Adolescent	Parents or guardian	Misattributed paternity	Patient portal	Avoiding care, delayed care, or family strife
Parent/Care Giver / Legal Guardian	Patient, other parent, or other care giver	Perinatally acquired sexually transmitted infection	Patient portal	Avoiding care, delayed care, or family strife
Parent/Care Giver / Legal Guardian	Patient, other parent, or other care giver	Substance abuse	Patient portal	Avoiding care, delayed care, or family strife
Parent/Care Giver / Legal Guardian	Patient, other parent, or other care giver	Parent or caregiver’s mental health	Patient portal	Avoiding care, delayed care, or family strife
Parent/Care Giver / Legal Guardian	Patient, other parent, or other care giver	Violence, abuse, or legal problems	Patient portal	Avoiding care, delayed care, or family strife
Parent/Care Giver / Legal Guardian	Patient, other parent, or other care giver	Misattributed paternity	Patient portal	Avoiding care, delayed care, or family strife
Parent/Care Giver / Legal Guardian	Patient, other parent, or other care giver	Stress associated with chronic care	Patient portal	Family strife or mistrust
Provider	Patient, other parent, or other care giver	Patient or family disagreement with provider	Patient portal	Delayed or missing documentation
Provider	Patient, other parent, or other care giver	Neglect or abuse	Patient portal	Lawsuit or unsafe environment for the provider

### Inadvertent Disclosure of Medically Relevant Information Obtained From Proxies

There may be situations in which health care providers may document pertinent information that they receive from parents, relatives, and legal representatives, which may adversely affect the patient, parent, or legal representative or damage relationships when disclosed (Table 1). For example, parents may disclose their difficulty in coping with an adolescent’s chronic illness to a provider who documents it in the adolescent’s chart. This information could then be seen by the adolescent in their patient portal and affect the parent–child relationship. Another example is if a parent discloses information about a drug use during pregnancy or perinatally acquired STI to the pediatrician caring for the newborn. This information would be accessible through the infant’s electronic record by other users, such as the other parent. In both situations, disclosing medically relevant information may be disincentivized for fear of its discovery by another person having access to the medical record.

### Health Care Provider–Patient Relationship in Difficult Diagnostic Dilemmas

Disclosure of information can adversely affect health care provider–patient relationships, especially when there is disagreement between the health care provider and the parents or patient (Table 1). In functional disorders where the medical work up does not demonstrate an organic etiology for the complaint, the parents or patient may believe otherwise. For example, when the defined Rome’s Criteria of Functional Abdominal Pain fits a patient’s symptoms, parents or the patient may disagree with this diagnosis. In similar cases where the relationship among the patient, family, and health care provider is critical to helping the patient improve, documenting this information could further damage a fraught or tenuous relationship with the health care provider. Although providers should hold themselves to high standards for documenting information in the EHR, providers should not feel pressured to augment their documentation for fear of their medical opinion offending patients or proxies. This can be the case when child abuse is in a differential diagnosis, and documentation of this in the child’s record may adversely affect the relationship between parent and health care provider if the parent feels unfairly accused or judged. There are situations where abuse is in a differential diagnosis, albeit with a very low index of suspicion, or where a provider may want to document that they have thought of but ruled out abuse or neglect. In these cases, it is unlikely the information will be compiled in “reasonable anticipation of, or for use in, a civil, criminal, or administrative action or proceeding” [32], which is clearly protected and eligible for legal blocking by HIPAA, and the remainder of the documented information may be of interest to the patient or proxy. The limited capabilities of data segmenting technology create an awkward or burdensome situation for providers.

## Older Adult Patients

### Overview

The complexity of care and the large number of comorbidities and treatments associated with aging make the electronic patient portal an attractive tool for persons with multiple health conditions. However, many older adults feel uncomfortable or ill-equipped using technology and rely on their caregivers for their health care–related tasks, necessitating proxy portal access. Less than 20% of US hospitals that allow caregiver proxy access also allow patients to filter or partially block the EHI passed on to their proxies [33]. Therefore, older adults are faced with many of the same challenges and potential harms that adolescents may experience.

### The Older Adult Patient–Caregiver Relationship

Despite an increase in STIs among adults over the age of 65 years, many older adults are reluctant to share a recent sexual encounter [34] with their health care provider, knowing that this information will be available to caregivers. Syphilis, which is a treatable condition, can mimic dementia and neurocognitive disorders in late stages of the disease if the diagnosis is missed. Similarly, older patients may withhold health information regarding mental health (including depression) and elder abuse (physical, sexual, emotional, neglect, abandonment, financial, and self-neglect) from their health care providers for fear of their proxy finding out (Table 1). Again, the emphasis on broad access may paradoxically erode the patient’s control over who can access their data. One potential solution may be allowing patients to block all information related to a specific topic from all users of the patient portal, including themselves, and unblock it again when they become sole users of the portal.

### Inadvertent Disclosure of Medically Relevant Information Obtained From Caregivers

Caregivers may disclose emotional, physical, or mental exhaustion leading to burnout. If this information is documented and shared with the older adult patient, unintended consequences are feelings of guilt, overburdening, and depression (Table 1).

Of note, there is a clause in the HIPAA Privacy Rule that specifically addresses keeping third-party information confidential. According to this clause [35]:

Any information disclosed to the provider by another person who is not a healthcare provider that was given under a promise of confidentiality (such as that shared by a concerned family member), may be withheld from the patient if the disclosure would be reasonably likely to reveal the source of the information.

Since the Cures Act defers to the HIPAA, this clause should be applicable under the Cures Act; however, this is likely not well-known or understood across institutions.

In some cultures, it is common practice for caregivers to withhold negative information such as the diagnosis of a cancer or a terminal illness. Caregivers are also frequently surrogate decision-makers and may for many reasons ask a health care provider to withhold a diagnosis [36]. Although we consider disclosure to the patient as the ethically preferable choice, we acknowledge that the inability to block information may not align with the cultural norms of certain patient groups [37]. The patient may also desire information blocking, such as when an older patient is afraid that disclosure of a new diagnosis of cancer or recurrence may burden their caregiver or lead to caregiver burnout.

### Health Care Provider–Patient Relationship in Difficult Diagnostic Dilemmas

Maintaining a good relationship with patients is critical for health care providers taking care of older adults, as dynamic shifts in health often require changing or transitioning goals of care. Even neutral personal descriptors such as “elderly” in a note can make patients feel judged and perceived themes of disrespect, errors, and surprises can lead to straining of a patient–provider relationship [38]. For example, the term palliative is often misinterpreted for end-of-life care when in fact the goal is symptom and quality of life improvement for any serious illness (even curative ones), irrespective of prognosis. Further, the National Center for Educational Statistics reports that 21% of adults in the United States (~43 million) are illiterate or functionally illiterate [39]. Misinterpreting documentation may prevent older adults from seeking care to relieve symptoms and stress and align treatment options with their goals. One unintended, but positive, consequence of the information blocking rules might be that it encourages providers to be more vigilant in their documentation to achieve language that is both medically accurate and affirming of the patient’s dignity.

## Where Information Blocking Went Too Far

Although the potential adverse outcomes previously discussed do not meet the Cures Act Final Rule definition of harm, in some cases, releasing this information may violate the foundational principles of a trusting provider–patient relationship.

### Information Blocking in the Multiuser EHR

While the Cures Act final has made it easier to access information electronically, this increase in access is not accompanied with the requisite technical advances to block access to data in appropriate circumstances. In the situations when information blocking can be legally used, strategies are limited in number and capability, especially in the context of a multiuser or proxy access. Information blocking is technically and logistically challenging, and the burden is placed upon the documenting health care providers to determine what EHI is and is not appropriate to block and to whom. For some health care providers, such as those practicing in adolescent medicine, family medicine, general pediatrics, pediatric subspecialties, internal medicine, and geriatrics, navigating information blocking may be a routine experience depending on patient needs.

At the institutional level, the hospital system can deactivate proxy access; however, this may be burdensome and can be delayed depending upon institutional implementation (eg, a health care provider clicking a button in the EHR versus contacting health information management and placing a ticket for a request to be completed). The ONC exceptions emphasize that information blocking should be no broader than necessary. It will be an infrequent occurrence that a patient or proxy is completely blocked from accessing all EHI and more common that the blocking will occur on a data-element-by-data-element (clinical documentation, laboratory tests, imaging, etc) basis. This may create a substantial burden for the health care institutions and be prone to user errors. Additionally, the absence of information may be conspicuous when a patient or proxy who usually receives information does not. There is an evolving standard called “Data Segmentation for Privacy” (DS4P) where a health care provider could mark portions of a note to be blocked from access; however, the adoption of this standard is minimal [40,41].

Beyond institutional policies and EHR technical capabilities, the health care provider can adopt new documentation workflows when information blocking is legally acceptable. For example, the health care provider could create one note that is appropriate to share with all users and another that includes the information which is then blocked (ie, shadow charting); however, this solution is time-consuming and burdensome and unlikely to be adopted as clinical documentation has already been shown to be a significant contributor to burnout among health care providers [42-46]. Further, duplicate documentation would also be error-prone, jeopardizing safety and creating additional work and confusion for other health care providers on the treatment team relying on documentation to support patient care. Health care providers may choose to avoid caring for patients who are more prone to these complicated situations.

Where information blocking is not acceptable, the health care provider, not wanting to damage a relationship or breach confidentiality, may decide to stop documenting certain information. This is a potentially dangerous practice that could affect medical care, reduce accurate billing, and result in incomplete communication about the patient’s medical history with other health care providers.

## Conclusions and Recommendations

The Cures Act Final Rule is undoubtedly necessary to facilitate significant improvements in patient care and innovation; however, in some cases of a multiuser or proxy access situation, the Information Blocking Provision conflicts with the standard that health care providers hold themselves to in the United States. Additionally, applying these exceptions to the Information Blocking Provision in legally acceptable cases will be burdensome and could lead to increased burnout among health care providers. Paradoxically, providing patients more control over their data may actually jeopardize their control and privacy in some scenarios. Although breaching confidentiality and damaging the patient–provider relationship will not necessarily cause substantial harm as defined by the text of the Final Rule, it may cause unnecessary anguish, limit the quality of care, or cause a patient to forgo or delay care, and lead to increased morbidity. Additionally, the privacy exception may be underutilized as it necessitates patients and providers be educated on the application of this rule and an institution’s policies and procedures. In light of these concerns, we recommend that ONC provide more detailed guidance both on their website and targeted outreach to health care providers caring for patients in the adolescent health setting and other multiuser or proxy access situations. As clause 171.202 (b) of the Cures Act allows institutions to develop policies around information blocking, we encourage ONC to develop and publish sample policies that institutions may use or modify. Such guidance should outline the exact processes by which a patient can opt out of their health data being shared with a proxy user using the privacy exception and detail how providers can best guide patients through decision-making without the fear of being in violation of the information blocking rules. Where data segmentation for privacy is not feasible, we recommend that ONC considers carving out an option for providers to return to traditional sharing options to prevent breaches of privacy. We also urge ONC to interpret the privacy exception broadly and not penalize hospitals or providers for information blocking when proxy access is the reason for the information blocking. We suggest that ONC and researchers carefully monitor charting habits in these multiuser or proxy access situations by studying how often patients use the privacy exception compared with single-user EHR access scenarios, how much time is spent documenting for these scenarios, and how much shadow-charting is taking place. We also suggest researchers carefully monitor the effect of information blocking on patient, provider, and proxy relationships. Additionally, we recommend limited penalties on health care providers in multiuser or proxy access situations during the implementation process of the Cures Act Final Rule until technological capabilities advance to better segment notes and block them from certain users.

## Abbreviations

DS4P: Data Segmentation for Privacy
EHI: electronic health information
EHR: electronic health record
HHS: US Department of Health and Human Services
HIPAA: Health Insurance Portability and Accountability Act
IT: information technology
ONC: Office of The National Coordinator for Health Information Technology
STI: sexually transmitted information
TAM: Technology and Adolescent Mental Well Being
